# Supplementary material for: Modeling Illustrates That Genomic Selection Provides New Opportunities for Intercrop Breeding
Source: Front Plant Sci. 2021 Feb 9;12:605172. doi: 10.3389/fpls.2021.605172 (PMC7902002; doi:10.3389/fpls.2021.605172)
Supplement: Supplementary file 2 [file Data_Sheet_2.zip › _IntercropSimulations_final/ReadMe.rtf]

Author: Jon BancicContact: jon.bancic@gmail.comModelling illustrates that genomic selection provides new opportunities for intercrop breedingDescription of simulations for intercrop breeding programs*** Data structure ***The file structure in this directory is the following: 	•	Three budget scenarios (i.e. BIG, MEDIUM, SMALL) were simulated for all five intercrop breeding programs (see main text of our publication). The main directory consists of 3 main folders named BIG, MEDIUM, SMALL.	•	Three correlation scenarios (i.e. 0.4, 0.7 and 0.9) between monocrop grain yield and intercrop grain yield traits were tested for all five intercrop breeding programs (see main text of our publication). Each main folder consists of subfolders named 04, 07 and 09.	•	Each subfolder (04, 07 and 09) consists of R scripts required to run the five intercrop breeding programs for each scenario.	•	There is an additional folder named ‘Analysis’ which consists of scripts for plotting, statistical analysis, and .rds files from our analysis.*** Simulation steps ***Each scenario can be broken down into:1. Burn-in breeding phase (script ‘BURNIN0X_Y.R’)	•	Creation of the founder populations for two component crops with specified correlation X between two traits	•	Twenty years of selection using phenotypic selection breeding program for specific budget 	•	This step is common for all future breeding programs2. Future breeding phase	•	Twenty years of future breeding using five intercrop breeding programs:	•	script ‘PHENO_Y.R’ for the phenotypic selection breeding program (Pheno)	•	script ‘CONVGS_Y.R’ for the baseline genomic selection breeding program (Baseline-GS)	•	script ‘GSPYT_Y.R’ for the preliminary yield trial genomic selection breeding program (PYT-GS)	•	script ‘GSDH_Y.R’ for the doubled haploid genomic selection breeding program (DH-GS)	•	script ‘GSGRID_Y.R’ for the Grid genomic selection breeding program (Grid-GS)*** To run simulations (an example) *** Simulations of each breeding program were run for each budget and for each trait correlation separately in 30 replications. Here, we give an example on how to run one replicate for the doubled haploid genomic selection program (DH-GS) with the medium budget and trait correlation of 0.9. Steps are the following:1. In R, enter the folder MEDIUM, then enter the folder 09 and set it as your home directory.2. Run the script ‘BURNIN09_M.R’ which will generate ‘BURNIN_M.RData’ file in your home directory.3. Run the script ‘GSDH_M.R’ which will load ‘BURNIN_M.RData’ file into R and simulate the future breeding phase using DH-GS breeding program. The script will generate ‘ResultsGSDH_S09.rds’ file which contains all reporting parameters.4. You can repeat step 3 and run a script for another breeding program (e.g. GSGRID_M.R) since the ‘BURNIN_M.RData’ file is the same for all five breeding programs within a replicate.5. The ‘.rds’ files can then be merged for plotting and comparison between breeding programs. See the script ‘PaperPlots_X.R’ for plotting and the script ‘ResultsAnalysis_Paper.R’ for statistical analysis in the folder ‘Analysis’. Note:	•	All simulations are computationally very demanding, take a long time to run and therefore therefore it is advisable to use a supercomputer	•	Make sure you have all required R packages installed (AlphaSimR, tidyverse, data.table, plyr, cow.plot, AlphaMME, Matrix).*** To plot results from our analysis *** Alternatively, you can try plotting results from our analysis using scripts in Analysis folder.In case the reader wishes to fully understand the details and dynamics of our simulations, we encourage you to contact us via the email provided above.
